# In vitro evaluation of composite resin fluorescence after natural aging

**DOI:** 10.4317/jced.56535

**Published:** 2020-05-01

**Authors:** Talissa-Mayer Garrido, Lidiane-Vizioli-de Castro Hoshino, Ronaldo Hirata, Francielle Sato, Antonio-Medina Neto, Victor-Hugo-Fazoli Guidini, Raquel-Sano-Suga Terada

**Affiliations:** 1 MS, DDS, Private practice, Maringa, Brasil; 2 PhD Student, MS, Department of Physics, State University of Maringa, Maringa, Brasil; 3 PhD, MS, DDS, Department of Biomaterials, New York University College of Dentistry, New York, United States of America; 4 PhD, MS, Department of Physics, State University of Maringa, Maringa, Brasil; 5 Graduate Student, Department of Dentistry, State University of Maringa, Maringa, Brasil; 6 PhD, MS, DDS, Department of Dentistry, State University of Maringa, Maringa, Brasil

## Abstract

**Background:**

Some composite resins contain luminophorous agents in order to reproduce tooth fluorescence. The objective of this study was to compare the fluorescence spectra emitted by composite resins with those of human enamel and dentin, and their emission behaviour after a 90-day natural aging period.

**Material and Methods:**

Nine shades of the composite resins Z350XT/3M (XT), Opallis/FGM (OP) and Empress Direct/Ivoclar-Vivadent (ED) were analyzed. Five specimens (10.0 mm x 2.0mm) were fabricated for each shade. Enamel (5.0 mm x 0.30 mm) and dentin (5.0 mm x 1.0 mm) specimens were obtained from sound human third molars. Fluorescence spectra of human dentin and enamel as well as the composite specimens immediately after fabrication were measured at the excitation peaks of 375, 395 and 410 nm. To assess composite resin fluorescence intensity changes over time, measurements were conducted after 30, 60 and 90 days, at 395 nm. Differences in fluorescence intensity over time were analyzed with ANOVA and Tukey’s test (*p*<0.05).

**Results:**

Fluorescence spectra baseline values of composites demonstrated no differences in intensity among the excitation peaks tested, with maximum emission found at the peak of 450 nm. Enamel and dentin spectra varied with different excitations, and the greater the excitation, the longer the wavelength in comparison to composite resins. After 90 days, XT presented an increase in fluorescence intensity, while OP and ED showed a reduction when compared with baseline values.

**Conclusions:**

Fluorescence intensity of composite resins changed during the period analyzed, with an emission behavior different from that of human enamel and dentin. The main changes occurred in the first 30 days.

** Key words:**Composite resins, dental materials, fluorescence, fluorescence spectrometry.

## Introduction

The human tooth presents the ability to emit visible light when exposed to ultraviolet rays, a phenomenon called fluorescence (1-4). Fluorescence intensity peaks for enamel are in the range of 450 to 470 nm (5-7), while for dentin, which has peaks three times greater than that of enamel, between 420 and 450 nm (3,7,8). As a result, teeth present a whitish-blue color (9-12), which make them appear lighter. Dynamic optical behavior of composites are mandatory to obtain a natural appearance during exposure to different sources of lights (10,13,14). Thus, the fluorescence of composite resins is fundamental in order to reproduce esthetic characteristics of teeth (15). Also, fluorescence of composite resins is important to contribute for the possibility to better differentiate resin from sound tooth in a substitution of failed restoration or in resin repairs (16) and to facilitate adhesive remotion used for bracket attachment after orthodontic treatment (17).

Composite resin manufacturers have incorporated luminophorous agents from rare earth metals in order to reproduce the phenomena of fluorescence. The most common metals used are europium, terbium, ytterbium and cerium (2-4,18-20). Clinically, fluorescence contributes to the aspect of vitality of the restoration, and helps to obtain the correct luminosity. When a non-fluorescent material is used, the aspect of the restoration tends to be impaired in the presence of ultraviolet light, such as those found in nightclubs and during the daylight (7,11,19).

Many studies have evaluated different fluorescence intensities of composite resins. It is already known that the shade and luminosity of composites tend to differ from those of the natural tooth, irrespective of the fluorescence intensity (2-4,11,19,20). However, most of the existing articles on the subject are not recent15 and there is a lack of studies evaluating the fluorescence of resin composites over time after natural aging. Also, the fluorescence spectra of composite resins compared to that of human enamel/dentin has not yet been investigated. So, this assessment can allow a qualitative evaluation of emission differences among the substrates, and provide a possible explanation for differences seen clinically.

Previous studies have evaluated fluorescence after accelerated aging, using ultraviolet light, changes in temperature, or water attack, and have demonstrated a variation in behavior between the different commercial brands (2-4,14,21-23). The fluorescence intensity values of some composite resins have been shown to increase (14), while others to diminish (21). Some factors may contribute to fluorescence emission, such as the composition (1,2,7) and type of composite resin (14,21). However, composite resins should maintain their properties over time (10,14,21). Thus, factors influencing fluorescence emission, as well as their mechanical behavior should be taken into consideration in the choice of material in order to ensure better clinical results.

Therefore, the aim of this *in vitro* study was to compare the fluorescence spectra emitted by different composite resins with those of human enamel and dentin, and to evaluate their emission behaviour after a 90-day natural aging period.

## Material and Methods

- Preparation of enamel and dentin specimens

A total of 20 enamel (5.0 mm x 3.0 mm x 0.03mm) and 20 dentin (5.0 mm in diameter x 1.0 mm) test specimens were obtained from healthy human molars, extracted for orthodontic reasons. To obtain the enamel test specimens, the buccal and palatal surfaces of the teeth were cut with a precision diamond disc (Isomet1000 - Buehler) and the dentin was removed with a spherical diamond bur N. 1016 (KG Sorensen), at high speed, under water cooling. For the dentin test specimens, approximately 3.0 mm of the occlusal portion of the teeth was removed, and after this the tooth was cut into slices in the transverse direction, resulting in 1.0 mm thick disc-shaped specimens. All specimens were verified with a digital caliper (Digimat Caliper, Mitutoyo Corp., Tokyo, Japan).

- Preparation of resin specimens

For the fluorescence evaluation of composite resins, 3 shades (achromatic enamel, chromatic enamel, and dentin) from 3 commercial brands (Table 1) were selected, totaling 9 groups, 5 specimens per group. Composite resin specimens were fabricated with the aid of a metal matrix measuring 10.0 mm in diameter and 2.0 mm thick, placed on a microscope slide and a polyester strip. Resin was inserted using a composite instrument N.1 (CIGFT 1, Hu-Friedy), taking care to avoid the formation of bubbles and excess of material. A nylon thread was placed in the center of the specimen so that it could be stored with no contact with the storage container.

**Table 1:**
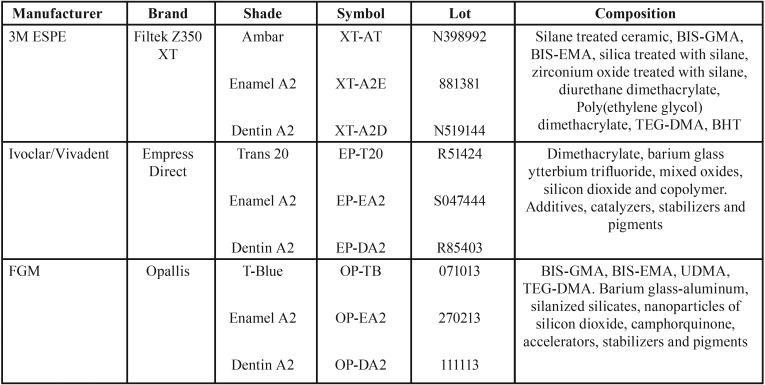
Manufacturers, brands, shades, lots and composition of resin composites used.

After filling the entire matrix, another polyester strip and microscope slide were placed on top and lightly pressed to allow the flow of excess material and obtain a smooth surface. Specimens were light polymerized with a Radii Plus (SDI) unit, for 40 seconds with the microscope slide kept in place, 20 seconds without, and another 40 seconds on the opposite side of the specimen, totaling 100 seconds. Then, specimens were removed from the matrix and marked so that all the readings were conducted on the same side. All specimens were produced by the same operator, under the same temperature and humidity conditions.

- Fluorescence evaluation

In order to obtain information on the behavior of the composites resins evaluated, excitation and fluorescence emission maps were drawn. Excitation was performed at every 5 nm, in the 300 to 420 nm range, and fluorescence emission readouts were obtained between 430 and 760 nm.

To compare the resin composite spectra with those of the dental substrates, human enamel and dentin, specimens were excited at 375, 395, and 410 nm always in the same position, immediately after the test specimens were obtained.

**Figure 1:**
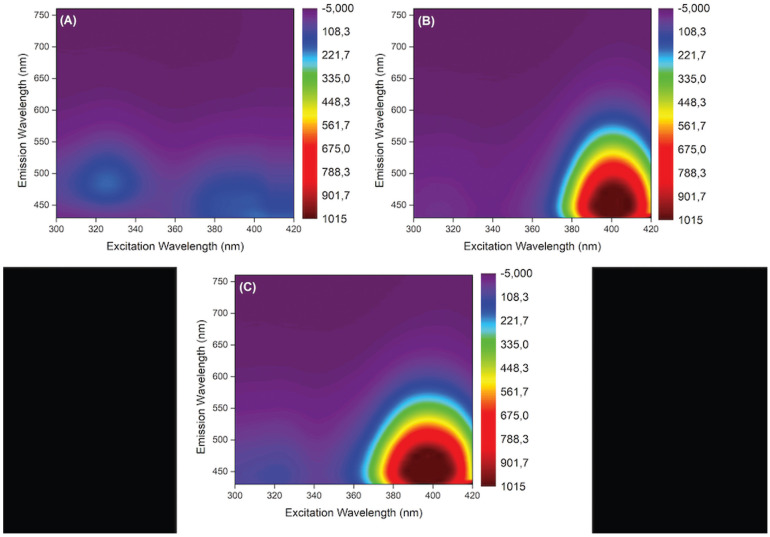
Emission (430 to 760 nm) and excitation (300 to 420mm) maps of the composite resins fluorescence evaluated: (A) XT-A2E, (B) OP-EA2, (C) EP-EA2.

To evaluate fluorescence over time, a fluorometer (PerkinElmer LS 55 Fluorescence Spectrometer) was used, and the fluorescence intensity of specimens of each shade was measured immediately after fabricating the specimens (baseline values). Then, specimens were stored in glass containers, immersed in 20 mL of distilled water, and stored in an oven at 37ºC. New measurements were conducted at 30, 60 and 90 days, in the same conditions. The distilled water in the containers was changed every month. For each resin composite specimen, spectra were obtained in 5 random positions, at the excitation wavelength of 395 nm, with the fluorescence emission readouts obtained in the range between 415 and 900 nm. Excitation was conducted at 395 nm, because the maximum fluorescence intensity of the composite resins evaluated occurred close to this wavelength (Fig. 1).

At baseline, resin composite specimens were excited at 375 and 410 nm for later comparison with enamel and dentin, and the fluorescence emission readouts were taken in the range between 400 and 900 nm.

All the readouts were normalized at double of the excitation used, at 750, 790 and 820 nm for the excitations 375, 395 and 410 nm, respectively. Spectra normalization was used to eliminate the intensity, reflection and scattering values that occurred during readouts.

- Data Analysis

Mean maximum intensity of the 5 positions measured was obtained for each shade for each excitation, after spectra normalization. Normalization was performed with regard to the second harmonic of the excitation wavelength (790 nm). Qualitative analysis of the resin composite spectra was conducted in comparison with the human enamel and dentin spectra. Descriptive statistics of the data was performed and ANOVA followed by Tukey test at a level of significance of 5%.

## Results

**Figure 2:**
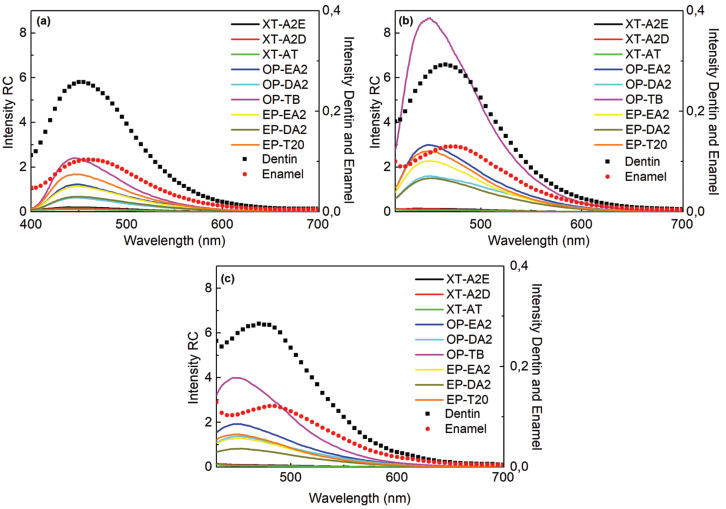
Fluorescence spectra of the composite resins, enamel and dentin in different excitations light: a) 375 nm, b) 395 nm and c) 410 nm.

Figure 2 shows the comparison of the fluorescence emission spectra of composite resins obtained at baseline, with human enamel and dentin at the excitations of 375, 395 and 410 nm. It may be noted that the behavior of composite resins did not change with the different excitations. However, fluorescence emission peaks of enamel and dentin were displaced slightly to the right as the excitation was increased.

**Figure 3:**
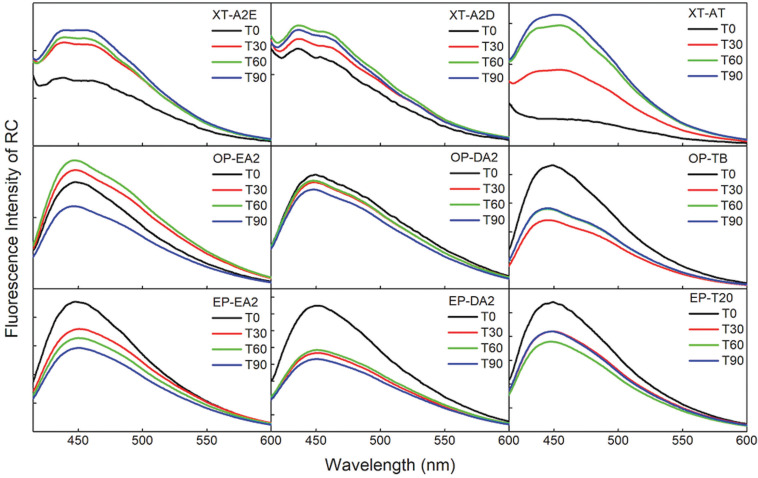
Spectras of fluorescence intensity variations of composite resins in different times: baseline (T0), after 30 days (T30), after 60 days (T60) and after 90 days (T90).

In general, over the 90-day test period, XT resin specimens presented an increase in fluorescence, while OP and ED resin specimens showed a reduction in comparison with baseline values (Fig. 3). The group that presented the highest fluorescence variation was XT-AT.

Mean fluorescence intensity variation for each material, among the evaluated time intervals can also be seen in Table 2. Statistically significant changes in fluorescence intensity after 30 days were observed in groups ED-T20, ED-EA2, ED-DA2, OP-TB, and XT-A2E. After 90 days, fluorescence intensity of OP-EA2, OP-DA2 and XT-AT changed significantly.

**Table 2:**
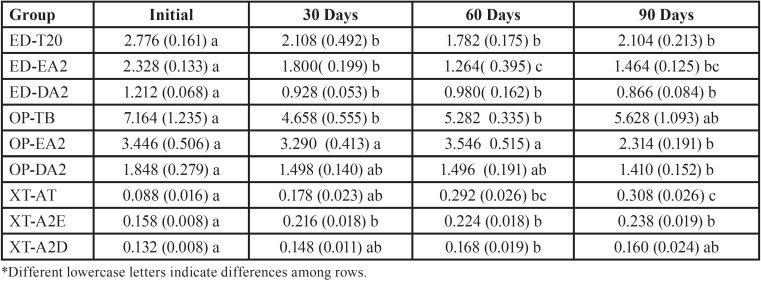
Means (standard deviation) of the variation in fluorescence intensity of composite resins between the time intervals evaluated.

Two shades of the Z350XT/3M Oral Care resin (XT-A2E and XT-AT), showed increased fluorescence intensity during the period analysed. All the others, except for OP-TB, showed diminished intensity.

## Discussion

Fluorescence spectra analysis of composite resins and dental substrate can contribute to explain the clinical differences in shade between the restoration and dental structure. The longitudinal evaluation of fluorescence permits to observe whether this property is maintained at esthetic parameters close to natural dental structures, and to show when these changes occur. The results of this study showed that the fluorescence spectra of human enamel and dentin undergo variations when excited at different wavelengths, which does not occur with resin composites. This was the first study that evaluated the spectra of fluorescence emission by composites and dental substrates under varied illumination conditions before.

The higher level of fluorescence of human dentin in comparison with enamel is due to the larger amount of organic materials present in the dentin, such as tryptophane and hydroxypyridine (3,7,25), which was also observed in this study. Analysis of enamel and dentin spectra in the present study demonstrated higher peaks of fluorescence for dentin. The emission peaks for enamel and dentin were at approximately 460, 470 and 480 nm for the excitations at 375, 395 and 410 nm, respectively, with the enamel curve slightly ahead of that of dentin for all the excitations. These results indicate that there is a difference in shade and luminosity emitted by composite resins and the dental substrate, and also in the behavior of the tested materials in response to different sources of lighting. Many composite resins tested in previous studies presented no fluorescence comparable to that of the tooth (7,11). The fluorescence of intact dentin has a shade closer to blue, whereas enamel and demineralized dentin have a whitish-blue shade. In our study, the fluorescence of all the shades of the tested composites tended towards the blue, differently from the luminosity bluish-whitish of a natural tooth (11).

All shades of resin composites tested (XT, OP AND ED) presented a significant variation in fluorescence intensity after 90 days. The maximum emission peak of composite specimens occurred at approximately 450 nm, which is in agreement with previous studies (1,7,8,24). Although other methods have been proposed for fluorescence analysis, such as spectrophotometers (1,2,4,8,10,14,21,22,24,26), direct spectrophotometry (13), and photographs (27,28), the spectrofluorometer is an appliance used to perform direct and reliable measurements of the fluorescence of solid bodies (7).

For the effect of aging on fluorescence emission, excitation of 395 nm was used because when the emission and excitation maps were analyzed, the maximum fluorescence intensity occurred close to this wavelength for all the composite resins. Other studies have also used similar wavelengths: 380 nm (13) or 398 nm (3). To analyze the behavior of fluorescence of the resin composites and dental substrates, excitations of 375 and 410 nm were also used, because these are the emission peaks of the “black light” mainly found in bars and night clubs, simulating different sources of lighting (15). This was also observed in an emission test performed previously with a black light lamp (Pl28 watts, 110 Volts).

Despite the fact that fluorescence is one of the most dificult optical properties to replicate artificially (11), dental materials manufacturers have incorporated rare earth metals into resin composites to produce this effect and different composites have been tested (2,18,29). However, the main fluorescent components of modern resin composites and method of incorporation are still unknown (2-4,19).

The results of the influence of the aqueous environment during specimen aging allowed the observation of the degradation of polymers of the organic matrix and the hydrolysis and enzymatic reaction mechanisms that promote oxidation and cleavage of the carbon chains (3,4,12). This would explain the reduction in fluorescence intensity of the composite resins OP and ED over time. In another study, a reduction of up to 65% in fluorescence intensity of the resin Opallis/FGM was observed (3). The luminophores may possibly have been chemically bound to the polymer chains and, therefore, the reduction in fluorescence may have been related to the polymerization process of the organic matrix in the first few days after light polymerization (3,10). Degradation of the organic complexes is found in composite resins over time, and as a result, bonds are broken and leached (4).

The behavior of XT group was the inverse to that of OP and ED; demonstrating increased fluorescence intensity as a function of time. A possible explanation may be related to changes in the organic component of this resin, and to the adequate polymerization of the material (10). Another possible explanation would be the “antenna effect”, in which the organic components absorb light and transmit it to the luminophores with the maturation of polymerization, considering that rare earth metals normally have low absorption (30). Possibly, for this particular brand of composite resin, greater light absorption occurred with aging, which led to fluorescence emission increase over time. In previous studies, the composite resin Z350XT/3M Oral Care, in the shade YT, and Filtek Supreme/3M Oral Care in the shades A2E and A2D, also presented low fluorescence that increased in intensity after aging (3,4).

Future studies should better analyze whether the relationship between luminescence, fluorescence and color exists and wheter the fluorescence intensity emitted corresponds exactly to the peaks of the same wavelength of the spectra of the tooth, i.e. of the two tissues combined, dentin and enamel. Also, since the incremental technique is the standard procedure, and the last layer is the most important for fluorescence behavior (13,19,26), investigations to verify if the material with very thin layers can maintain the same fluorescence characteristics are required.

## Conclusions

The fluorescence spectra of the three brands of composite resins at the excitations of 375, 395 and 410 nm were similar, with maximum emission peak at 450 nm. The enamel and dentin spectra varied at the excitations of 375, 395 and 410 nm, and the greater the excitation the greater the wavelength in which they presented peaks of fluorescence emission. There was a significant variation in fluorescence intensity among the resins analyzed, during the period of 90 days. In general, XT presented an increase, while OP and ED a decrease in fluorescence intensity in comparison with baseline values. The main changes occurred in the first 30 days.
